# Investigation of Potential Use of Soybean Protein Isolate–Chinese Bayberry Tannin Extract Cross-Linked Films in Packaging Applications

**DOI:** 10.3390/ma15155260

**Published:** 2022-07-29

**Authors:** Jingjing Liao, Shuangqi Deng, Hisham Essawy, Xiaoyan Bao, Hongyan Wang, Guanben Du, Xiaojian Zhou

**Affiliations:** 1 Yunnan Provincial Key Laboratory of Wood Adhesives and Glued Products, Southwest Forestry University, Kunming 650224, China; seventwodeng@163.com (S.D.); y244261076@163.com (X.B.); gongben9@hotmail.com (G.D.); 2 National Forestry and Grassland Administration Key Laboratory of Plant Fiber Functional Materials, Fuzhou 350108, China; 3 National Research Centre, Department of Polymers and Pigments, Dokki, Cairo 12622, Egypt; hishamessawy@yahoo.com; 4 Zhejiang Academy of Forestry, Hangzhou 310023, China; 15990054143@163.com

**Keywords:** soybean protein isolate, Chinese bayberry tannin, cross-linking, antioxidant

## Abstract

The possibility of using commercial bayberry tannin (BT) from a Chinese source as a cross-linker and functional additive to develop soybean protein isolate (SPI)-based films was explored in this study by using the solvent casting method. In particular, the impacts of BT loading on the tensile strength, microstructure, thermal stability, water resistance and antioxidant capacity were fully investigated. The results reveal that SPI incorporated with BT yielded a phenolic–protein hybrid whose relevant films exhibited an improvement in tensile strength of around two times greater compared with native SPI as a result of the formed interactions and covalent cross-links, which could be proven using FTIR spectroscopy. The introduction of BT also led to the compact microstructure of SPI–BT films and enhanced the thermal stability, while the water vapor permeability was reduced compared with the control SPI film, especially at high loading content of tannin. Additionally, the use of BT significantly promoted the antioxidant capacity of the SPI-based films according to DPPH radical scavenging assay results. On this basis, Chinese bayberry tannin is considered a promising natural cross-linker and multifunctional additive that can be dedicated to developing protein-derived films with antioxidant activity for food packaging applications.

## 1. Introduction

Food packaging presents an extremely vital role in safely distribution and prolongation of shelf-life in today’s society and supply chains. Unfortunately, most food packaging made from non-renewable resources is designed to be disposable and less valuable to recycle. As a consequence of thriving e-commerce and the COVID-19 pandemic, the rapidly increasing demand for packaging material has intensified pressure on earth’s ecosystems in recent years [1,2]. This leads to the urgent need to develop renewable and degradable food packaging from biopolymers such as proteins, lipids and polysaccharides [3,4,5], reducing the negative impact on our environment.

Various proteins, such as zein [6,7], casein [8], whey protein [9,10], wheat gluten [9,11,12] and soy protein [4,13], are considered as promising biopolymers for employment in food packaging films because of their favorable film-forming capacity and impressive gas barrier properties compared with those prepared from lipids and polysaccharides [4]. In particular, soy protein isolate (SPI), a by-product of the soybean oil industry, is showing the greatest price advantage compared to other proteins, including resource abundance, large manufacturing scale and high protein concentration (>90%) [4]. SPI films can be easily prepared by drying cast protein solution since the buried functional groups of protein, such as hydrophobic and sulfhydryl groups, are easily exposed through heat treatment. Film networks are then formed through hydrophobic interactions and disulfide bonds of these exposed functional groups [14]. Besides, SPI-based films present good biodegradability [4], which can be easily degraded in 14 to 33 days under indoor soil burial conditions as reported by González et al. [3]. However, the use of SPI-derived films for packaging applications is still restricted due to their poor mechanical properties, weak resistance to water vapors and thermal stability, all of which are due to the hydrophilic nature of proteins and their weak interactions within film networks. Great efforts have been exerted to mitigate these drawbacks, via physical, chemical or enzymatic treatments, incorporation with hydrophobic additives and use of cross-linkers. Among them, cross-linking modification is considered as the most efficient strategy as cross-linkers can react with active groups of protein, forming covalent bonds and enhancing the inter- and intramolecular structure of film networks [13]. In past decades, the most extensively used cross-linkers have been formaldehyde, glutaraldehyde and glyoxal due to their cross-linking efficiency and low cost, but the cytotoxicity of these aldehydes limits the application of SPI-derived films in the food packaging field [15]. To overcome this challenge, safe cross-linkers from renewable resources have been widely explored to determine their ability to cross-link with SPI. However, the reported cross-linkers, such as transglutaminase [16,17] and genipin [18], present a smaller price advantage with regard to industry applications.

**Figure 1:**
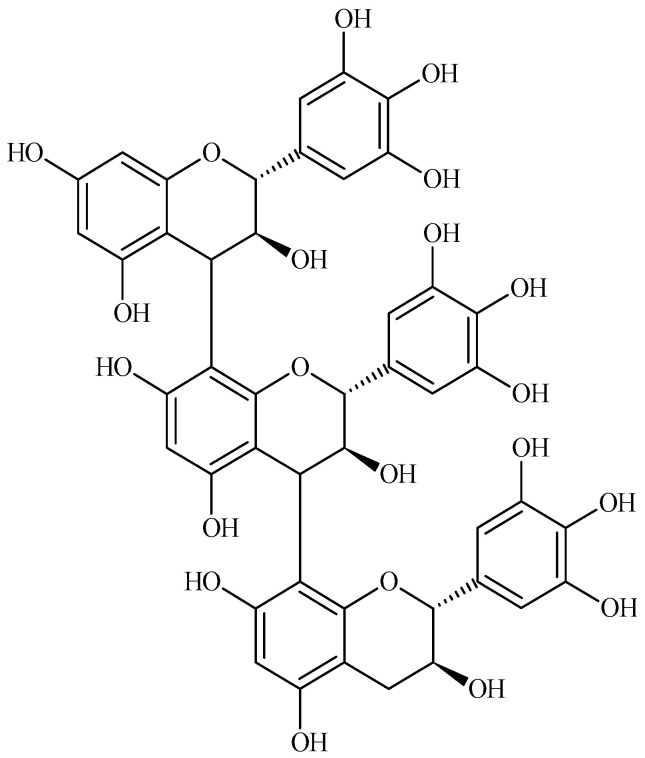
Structure of prodelphinidin.

In recent years, tannins have garnered great interest in protein-based food packaging due to their strong interaction with proteins, outstanding antioxidant action, resistance to ultraviolet (UV) radiation and antifungal and antimicrobial properties [19,20]. Tannins are generally phenolic compounds with molecular masses from 500 up to 3000 Da that are highly abundant in almost all parts of plants (e.g., wood, barks, leaves, roots, fruits and seeds). They are structurally divided into hydrolysable and condensed types. Various tannin types were reported to advance the performance of films formulated from proteins, such as tannins extracted from grape seed, green tea, blueberry and cranberry [21,22,23], tannin acid [23,24,25], ferulic acid [24,26], valonea tannin [27], chestnut bur extracts [28,29] and wood tannin extracts [30]. Enhanced mechanical strength and improved water vapor permeability, and special functions such as anti-UV and antioxidant and/or antibacterial capacities, were investigated in these reports owing to the chemical activity and bioactivity of tannins. For instance, SPI-based film incorporated with 10% valonia tannin results in nearly 33% improvement of tensile strength, decrease by 13% of water vapor permeability and 80% of oxygen permeability compared with native SPI-based film, owing to the cross-linking function of tannin [27]. Cao et al. reported SPI cross-linked by 4.0% ferulic acid with the help of cellulose nanofiber can significantly delay the lipid oxidation process of pork owing to the free-radical scavenging ability and cross-linking capacity of ferulic acid, blocking water, oxygen and ultraviolet light [24]. Among these reports, wood tannin extracts were particularly promising for building cost-effective packaging material because they can be easily extracted from forestry residues and wood industry wastes, and constitute above 90% of the global commercial production of tannins [31]. Traditionally, tannins produced from either wood or tree bark can be utilized in the process of leather tanning and in wood adhesive formulations as well because of their good chemical reactivity and low price [32]. A recent review reported the use of wood tannins as additives for food, given that such phenolic structures are cost-effective, stable during processing, have an increased shelf-life, are friendly to food stuffs and show good efficiency even at small concentrations [19]. Moccia et al. reported that condensed tannins from Quebracho tree bark tend to be more stable and have superior antioxidant properties compared with hydrolysable tannins in the application of antioxidant coatings for nylon filters [30]. Moreover, wood tannin extracts bearing a high molecular weight are likely to pursue a strong interaction with proteins, resulting in better thermal stability and mechanical properties [11]. Chinese bayberry (*Myrica rubra Sieb. Et Zucc.*), a subtropical evergreen fruit tree, is native to China and is now also widely grown in other Asia countries [33,34]. The high medicinal value of Bayberry leaves and barks has been recorded in the book of Chinese herbal medicine, and their biological activity has been widely studied since Chinese bayberry extracts contain a high amount of tannins with an excellent antioxidant capacity [35]. Tannin from bayberry bark is one of the most common commercial tannins in China, mainly consisting of prodelphinidin structures ranging from dimers to tridecamers (Figure 1). However, few studies have attempted to explore its possible innovative applications. 

Our previous studies have shown that Chinese bayberry tannin (BT) presented a good antioxidant function of BT incorporated into PVA films [36,37]. However, the imperfect miscibility and weak covalent bonds of two components were generally found, even with the help of aldehydes, due to the large molecular weight of BT, which makes its incorporation into PVA more difficult compared with other polyphenols. In order to achieve the optimal performance of BT in packaging film, SPI was chosen as a green matrix in this work on account of its strong interaction with high-molecular-weight tannin and SPI [20,38]. The effect of various BT loading contents on the cross-linking capacity of SPI films was evaluated in terms of the mechanical, morphological and thermal characteristics and permeation of water vapors, along with the antioxidant capacity of the films.

## 2. Materials and Methods

### 2.1. Materials

Food-grade soybean protein isolate, with a protein content of more than 90%, was purchased from Ruikang Biological Technology Co., Ltd. (Yueyang, China). Chinese bayberry tannin (72%) with an average molecular weight of 2292 Dalton [34,39], was obtained from Guangxi Wuming Company (Nanning, China). Analytical-grade reagents such as glycerol, sodium hydroxide and anhydrous calcium chloride were provided by Tianjin Fengchuan Chemical Reagent Technology Co., Ltd. (Tianjin, China), Chengdu Kelong Chemical Reagent Factory (Chengdu, China) and Guangdong Guanghua Technology Co., Ltd. (Shenzhen, China), respectively. 1,1-diphenyl-2-picrylhydrazyl (DPPH, 96%), a widely used reagent for the radical scavenging assay, was purchased from Shanghai Macklin Biochemical Co., Ltd. (Shanghai, China).

### 2.2. Film Preparation

A solution of soy protein isolate (SPI) (6%, *w*/*v*) was prepared by dissolving 6 g in 100 mL of distilled water under magnetic stirring. Sodium hydroxide solution (NaOH) at a concentration of 2 mol/L was used to adjust the pH to 11. Various loading contents of BT (0%, 5%, 10%, 15%, 20%) based on SPI solid content and 3 g of glycerol were mixed with the SPI solution and stirred for 10 min. After that, the mixture was moved to a round-bottomed flask and placed in a water bath at 85 °C under magnetic stirring. After 20 min of reaction, around 40 mL of film-forming solution was then used for casting films on a Teflon plate with a size of 16.6 mm × 26.6 mm, followed by air drying at room temperature. Lastly, the films were peeled off very carefully and transferred to a desiccator with 50% humidity for more than 24 h before further use. The obtained films were termed ST0, ST5, ST10, ST15 and ST20 based on their BT loading content. All films presented a similar average thickness of 0.12–0.15 mm. 

## 3. Characterization

### 3.1. Fourier Transform Infrared (FTIR)

The interaction between SPI and BT was tracked with an attenuated total reflection (ATR) FT-IR spectrometer (Thermo scientific Nicolet IS50). An average of 32 scans were undertaken for each sample between 600 and 4000 cm^−1^ with 4 cm^−1^ resolution. 

### 3.2. Tensile Strength

A universal testing machine (model 5569) was utilized for evaluating the tensile stress and elongation at rupture of SPI–BT films according to GB/T 1040.3-2006. The test conditions were a temperature of 23 °C and a relative humidity of around 45–55%. The rectangular-shaped specimens (20 × 160 mm) were uniaxially deformed with a speed of 50 mm/min and a grip separation of 50 mm. Five repetitions were employed to calculate average values and standard deviation.

### 3.3. Opacity

Each tested sample (4 cm × 1 cm) was initially put in the cell of a UV spectrophotometer (WFZUV-8802S), and the absorbance at 600 nm was recorded. Opacity values were calculated using Equation (1) [40]:

Opacity = Abs_600_/x (1)

where Abs_600_ is the sample absorption at the defined wavelength, and x is the sample thickness.

### 3.4. Thermal Gravimetric Analysis

The thermal gravimetric analysis (TGA) was achieved using a thermoanalyzer (TG209F1) for investigating the thermal behavior of BT-cross-linked SPI films. All tested samples (5–10 mg) were heated over a temperature interval from 30 to 600 °C at a rate of 10 °C/min under nitrogen protection. 

### 3.5. Morphology

The morphology of each sample was examined over a cross-sectional area with a scanning electron microscope (SEM, Zeiss Sigma 300) using an accelerating voltage of 5 kV after prior freezing in liquid nitrogen to induce fracture. A cross-section of the fractured area from each sample was gold-sputtered prior to observation.

### 3.6. Permeability to Water Vapor (WVP)

The permeability of SPI–BT films to water vapors was evaluated using the cup method with minor modification according to the work of Wen and Yong [40,41]. Briefly, 3 g of anhydrous calcium chloride was charged to a beaker with an inner diameter of 50 mm and a height of 70 mm. Each beaker was covered by the resulting films with the help of a parafilm and transferred to a desiccator filled with 1000 mL of distilled water to provide 100% relative humidity. Each beaker was weighed twice per day over three successive days. The WVP was determined according to Equation (2):

(2) $$\mathrm{WVP}\;(g/s\cdot \mathrm{mm}\cdot \mathrm{pa})=\frac{W\times x}{A\times \Delta P\times t}$$

where W is the increase in weight for each beaker (g), x is the thickness of the film (mm), t is test time (s), A is the area of permeation for each sample (mm^2^), and ΔP is the partial vapor pressure (2063.79 Pa at 18 °C).

### 3.7. Antioxidant Activity

The radical scavenging assay of DPPH was studied to estimate the antioxidant activity as previously reported in our previous works [36,37]. Initially, 0.1 g of the tested sample was immersed in 30 mL of distilled water while magnetic stirring was applied for 30 min. Afterwards, 1 mL was withdrawn from the solution and mixed with 4 mL of DPPH–methanol mixture (150 µmol/L) and kept in the dark for an additional 30 min. Similarly, 1 mL of distilled water and 4 mL of DPPH–methanol mixture were mixed and kept in the dark for 30 min as a control sample. An ultraviolet spectrophotometer (WFZUV-8802S) was utilized for measuring the absorbance of the mixtures at 517 nm, and the scavenging action was evaluated with Equation (3):

(3) $$\mathrm{RSA}\;(\%)=\left[1-\frac{A_{sam}}{A_{con}}\right]\times 100$$

where *A_sam_* and *A_con_* represent the relevant absorbance of the sample and control, respectively. Each assay was repeated three times.

## 4. Results and Discussion

### 4.1. The Reaction Mechanism of SPI-BT Films

**Scheme 1:**
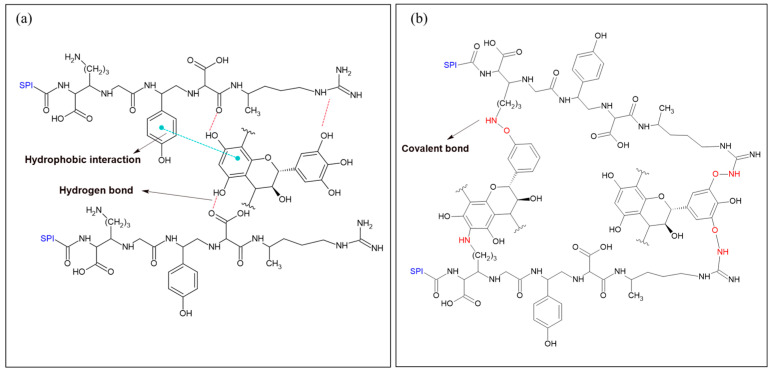
Possible chemical bonding between SPI and BT in resulting films: (**a**) non-covalent bonds; (**b**) covalent bonds.

The interaction between tannins and proteins involves extensive hydrogen bond formation between hydroxyl groups from tannins and the amide groups from proteins, along with hydrophobic interactions between the aromatic rings of tannins with hydrophobic regions and hydrophobic amino acid residues [12]. As expected, under alkaline conditions and a relatively high temperature, the native disulfide bonds of SPI were cleaved, exposing hydroxyl, amino and carboxylic acid groups. Meanwhile, phenolic rings of BT underwent oxidation to form ortho-quinone structures, which increased the cross-linking liability with peptide chains and the formation of new covalent bonds with SPI molecules, mainly through the Schiff base reaction and Michael addition [38,42]. The ongoing interactions between SPI and BT are simplified in Scheme 1, which was developed with the help of ChemSketch (freeware, file version C25E41).

**Figure 2:**
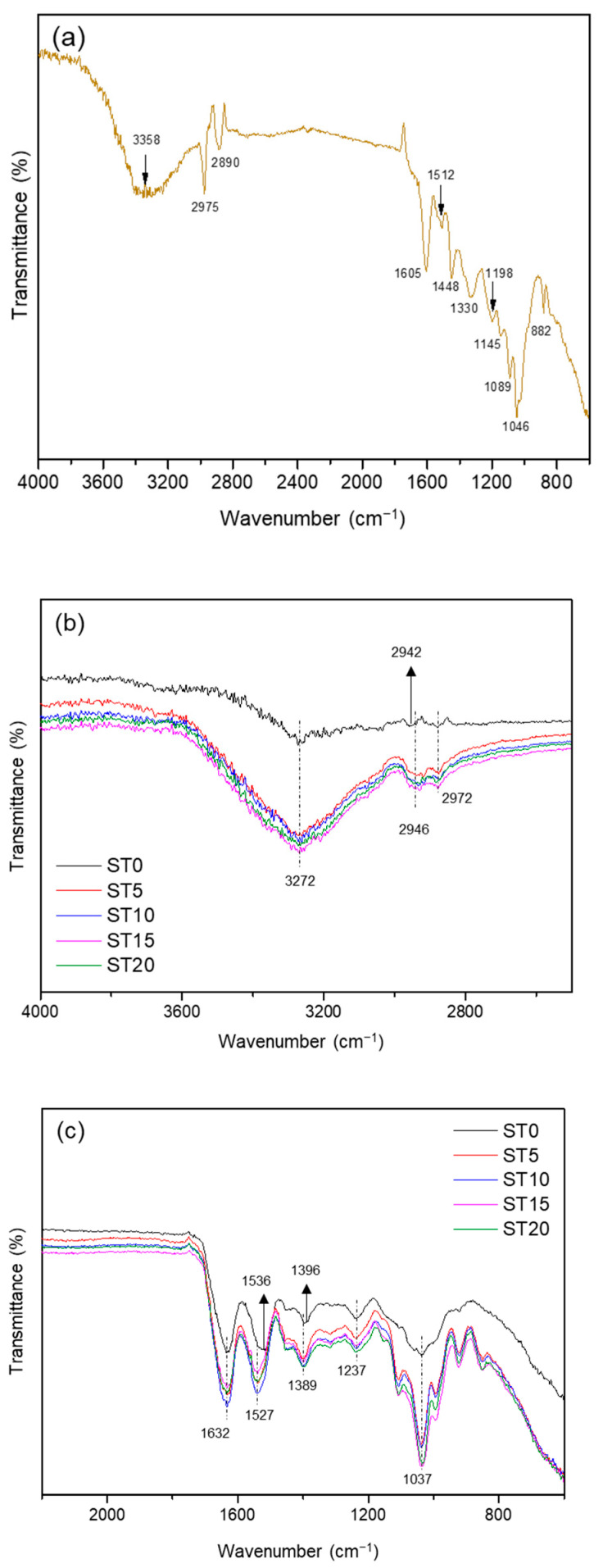
FTIR spectra of (**a**) BT and (**b**,**c**) SPI–BT films.

The chemical interactions between SPI and BT were investigated using FTIR. It can be observed from Figure 2a that the spectrum of tannins exhibited an expanded peak centralized at 3358 cm^−1^, which can be assigned to stretching vibrations of the hydroxyl groups, as well as the bands of 2975 cm^−1^ and 2890 cm^−1^, pertaining to the stretching of C–H. The characteristics of the aromatic skeleton (1605 cm^−1^ and 1512 cm^−1^), aromatic ring vibration (1448 cm^−1^), C–O stretching of the aromatic ring (1330 and 1198 cm^−1^) and C–O of methoxyl groups (1046 cm^−1^) and C-H out-of-plane bending (aromatic ring) were also detected. The high intensity of the band at 1605 cm^−1^, related to C–C stretching, indicates the dominance of C4–C8 interflavonoid linkages in BT [43]. Figure 2b,c reveal the spectra of a native SPI film (ST0) and SPI films containing various ratios of BT. In the case of the native SPI film, a strong band at 3272 cm^−1^ was derived from N–H and O–H of amide A. The characteristic absorption peaks at 2942 and 2872 cm^−1^ are ascribed to CH_2_ and CH_3_ stretching. Three amide absorption peaks recognized at approximately 1632, 1527 and 1237 cm−1 indicate C–O stretching (amide I), N–H deformation (amide II) and C–N stretching and N–H vibration (amide III), respectively [42]. Bands at 1389 cm^−1^ and 1037 cm^−1^ are indicative of COO– and C–O. As was noticed in the spectra of SPI–BT films, the peak revealing phenolic –OH groups of tannin, appearing typically at around 3358 cm^−1^, disappeared, and the absorption band of amide A (3272 cm^−1^) became wider with more additions of BT, suggesting that weakened O–H bonding vibrational energy and phenolic hydroxyl groups are involved in the interactions between SPI and tannin [44]. In addition, the vibration peak of CH_2_ shifted, the peak intensity of amide I increased, the vibration peak of amide II changed from 1536 cm^−1^ to 1527 cm^−1^, and the absorption band of COO– moved from 1396 cm^−1^ to 1389 cm^−1^. These perturbations can be correlated with SPI–BT cross-linking via hydrogen bonding between hydroxyl groups on the tannin rings and amino groups of SPI, as well as covalent linkages between oxidized quinone rings of BT and free amino groups from SPI [13,33,44], all of which are in compliance with previous findings on cross-linking of SPI [45] and casein [8] by phenolic acids. The combination of peaks related to BT and corresponding peaks of SPI in addition to the intensive broadening at the OH and amide regions confirms that the interaction between tannin and SPI occurred via the contribution of these sites. This can be further evidenced by the intensification of other peaks pertaining to SPI with more additions of BT as a cross-linker.

### 4.2. Strength Evaluation

The strength and flexibility of ST0 and SPI–BT films with various tannin contents were evaluated using tensile stress (TS) and elongation-at-failure (EB) measurements. Table 1 indicates that SPI–BT films incorporated with BT acquired more than a two-fold increase in tensile strength compared with control SPI films (ST0), suggesting that weak protein–protein interactions (hydrogen bonding, hydrophobic interactions and S–S bonding) were strengthened by protein–phenolic cross-linking interactions. Meanwhile, the elongation at the break of SPI–BT films showed a decreasing tendency because of the cross-linking in the network, which greatly restricted the molecular mobility of SPI chains. Nevertheless, the obtained elongations at the break still qualify these films for use in packaging applications. Similar findings were reported for many protein-based films incorporating tannins, such as gluten films with tannin acid and apple tannin [11], caseinate and gelatin films with condensed tannins from grape or oak barks and SPI films with valonea tannin [29]. It was noticed that the films containing 10% BT achieved maximum tensile stress, which is in line with previous reports described elsewhere [29,46], revealing the intermolecular interactions between SPI and BT reached a maximum at a certain concentration and would not develop more with more additions.

### 4.3. Physical Appearance, Opacity and Morphology

Physical appearance and opacity are critical properties of films dedicated to packaging applications. The relevant data for SPI–BT films were collected and are presented in Table 1. The native SPI film appeared generally light-yellowish with a comparatively good opacity value. However, the color of SPI–BT films showed a brownish appearance with increased opacity values. The transparency of SPI–BT films was reduced with increasing BT content. Similar trends have been described in studies of SPI films containing different tannins [13]. The brownish color is ascribed to the inherent color of tannins, while the reduced transparency might be a result of the formation of a denser film surface as a result of the strong interaction between SPI and BT.

**Figure 3:**
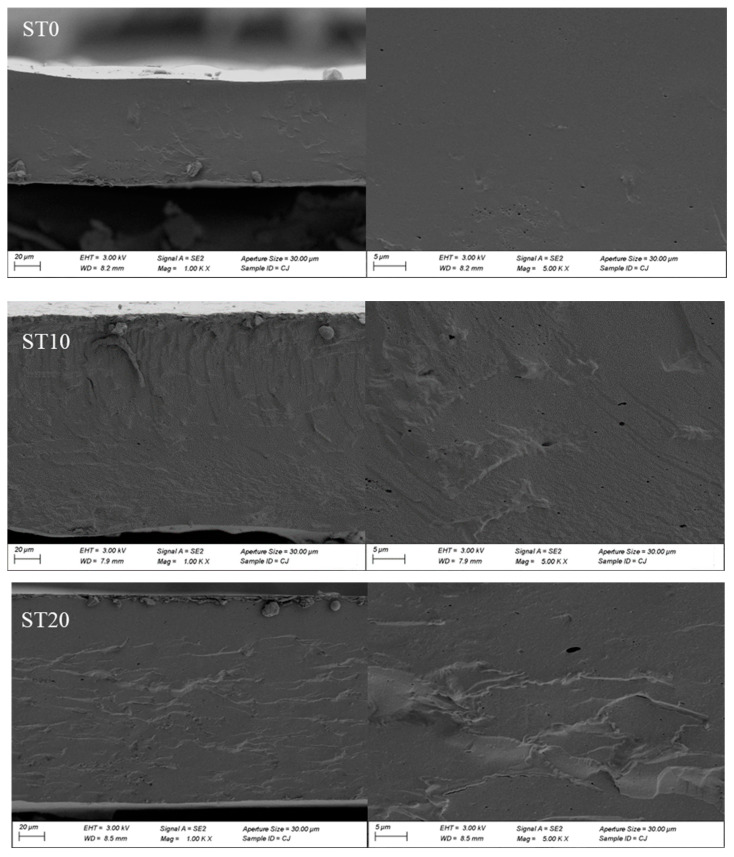
The SEM fractured cross-section micrographs of SPI and SPI–BT films.

Cross-sectional microstructures of ST0, ST10 and ST20 were observed using SEM microscopy, and their relevant micrographs at two different magnifications are displayed in Figure 3. The fractured surface of ST0 shows smoothness and uniformity, while the fracture surfaces of SPI–BT films became denser and rougher with coarse textures, indicating intensive interactions are involved in the construction of complicated networks combining SPI and BT.

### 4.4. Thermal Stability

**Figure 4:**
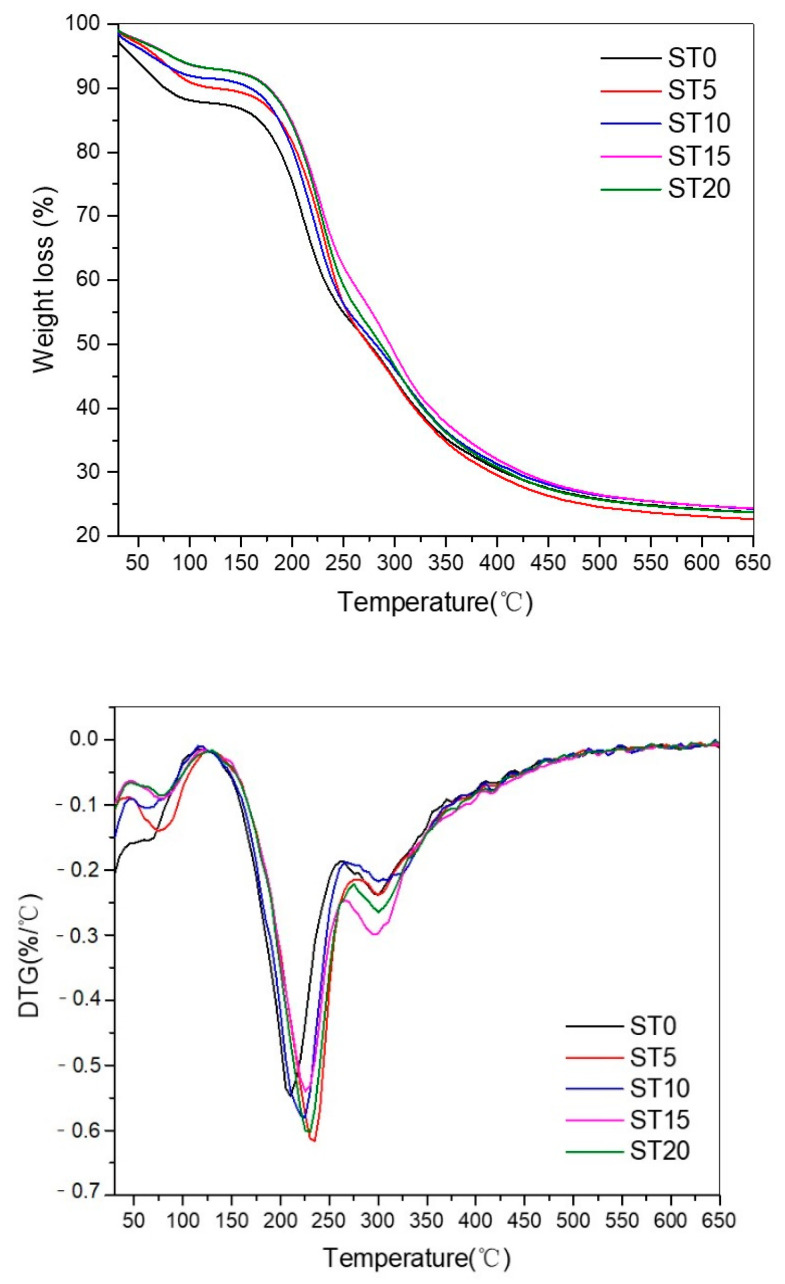
TGA and corresponding DTG traces of SPI and SPI–BT films.

The thermal stability of SPI–BT films was investigated using TGA, and the corresponding weight loss and derivative thermogravimetric (DTG) profiles were recorded in the range of 30–650 °C, as demonstrated in Figure 4. All films displayed a similar response to thermal actions, from which a three-stage weight loss process can be described according to the DTG curves. The first stage was undertaken with approximately 10% weight loss in the range of 80–125 °C, associated with the liberation of absorbed and bound water. The second interval of weight loss occurred over 150 to 270 °C, corresponding mainly to breakage of the ties between protein chains of SPI with BT [47], which was corroborated by a forward shift of around 40 °C of this degradation peak with respect to the neat SPI film and the development of increasing broadness with a greater content of BT. The third stage could be correlated to the rupture of the main protein skeleton and the decomposition of peptide bonds [38]. Thus, the transfer of the temperature associated with the highest degradation rate of the films incorporated with BT compared with ST0 suggests enhanced thermal stability due to the ongoing interactions between SPI and BT. It is obvious that the extent of enhancement is related to the types of protein and tannin together with the conditions of their interaction including pH and temperature [48].

### 4.5. Water Vapor Permeability (MVP)

**Figure 5:**
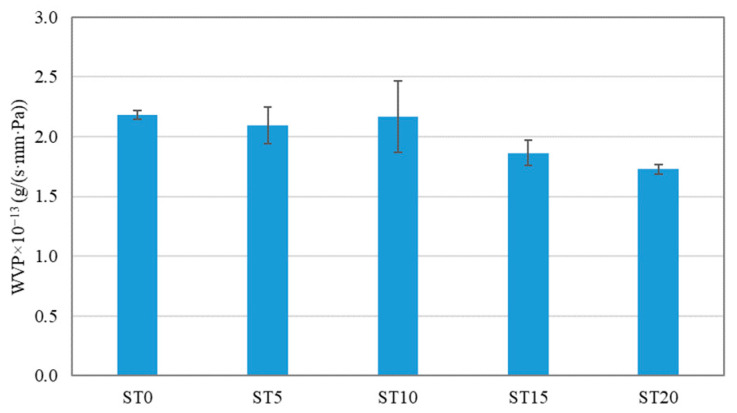
The WVP of SPI and SPI–BT films.

WVP is an important parameter revealing the moisture transfer between food and the surrounding environment, which relies on intermolecular interactions of SPI and tannin as well as the microstructure of the resulting films. The WVP of SPI films with different BT loading contents is given in Figure 5. The WVP of the films containing less than 10% BT was comparable with ST0, while that of SPI–BT films with higher BT loadings was decreased. In general, the interaction between protein and tannins results in a compact structure, which decreases the transmission of water molecules across the films. However, the WVP of protein–tannin films also depends on hydrophilicity and cross-linking extent of tannin [48]. For example, SPI films incorporated with valonea tannin showed increased WVP [27], while reported myofibrillar protein films incorporated with grape seed tannin or apple tannin presented a decreased WVP [21,48]. In correlation with FTIR characterization and the denser microstructure of ST20 as revealed from imaging with SEM microscopy, the intensive interaction of BT with SPI via hydrogen bonding, hydrophobic interactions and covalent bonding, in addition to the reduced free volume of SPI with increasing BT content, caused more tortuosity and a longer pathway for water molecules to go through the SPI–tannin network [21]. Thus, SPI–BT films exhibited a trend of decreasing WVP with increasing BT loading.

### 4.6. Antioxidant Activity

**Figure 6:**
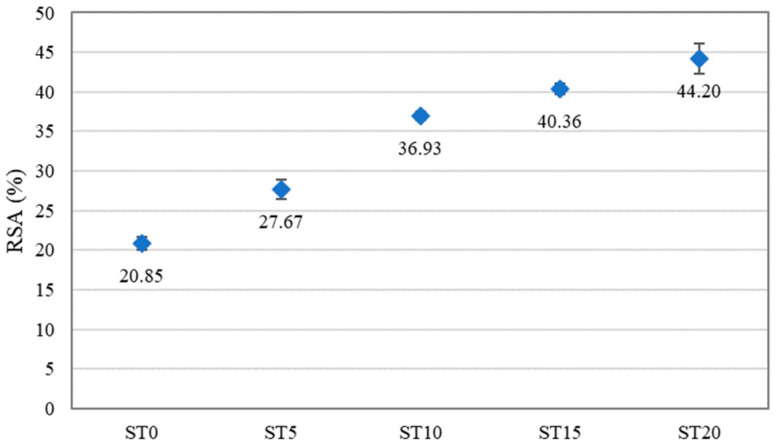
DPPH radical scavenging performance of SPI as well as SPI–BT films.

The DPPH assay, a standardized method for evaluating the antioxidant capacity of materials, was employed to evaluate the antioxidant activity of the films, and the results are presented in Figure 6. With the presence of the peptide fraction in SPI, the native SPI film exhibited 20.85% DPPH radical scavenging activity, as widely reported for protein-based films [27,29]. The SPI–BT films exhibited an obviously enhanced DPPH scavenging capacity, especially in the case of the films loaded with a high BT content. This phenomenon could be correlated with the presence of weak interactions via hydrogen bonding between SPI and BT, causing the tannin to be easily liberated from the films. Undoubtedly, BT has a good effect on the strength of SPI-formulated films along with promoting their antioxidant capacity. Therefore, BT is considered a promising alternative and safe cross-linker and antioxidant additive for protein-derived films.

## 5. Conclusions

Tannins from Chinese bayberry barks can be employed effectively for developing SPI films with enhanced performance. The introduction of BT with SPI caused the strengthening of films derived from the produced SPI–BT hybrid, which can be ascribed to various developed cross-links established between SPI and BT. The incorporation of BT was not only beneficial for the cost efficiency, being environmentally friendly due to its bio-sourced materials, but also imparted upgraded thermal stability and strength under optimized conditions. Further, the introduction of BT was responsible for the acquired compact microstructure and lower WVP because of its tendency to produce a high extent of cross-linking and the hydrophobicity of its monomeric units. Consequently, this can be associated with a reduction in free volume, producing network structure and increasing the passageway of vapors inside the films. More interestingly, the property of a high antioxidant capacity, which BT is known for, was transferred to the SPI-based films, indicating that some free sites on BT were still available to contribute to the antioxidation activity. Therefore, Chinese barberry tannin is often applied as a cross-linking and multifunction additive, especially in packaging applications.

## Figures and Tables

**Table 1:** Mechanical properties, optical appearance and opacity of SPI–BT films.

	TS (MPa)	EB (%)	Optical Appearance	Opacity
ST0	0.94 ± 0.06	247.69 ± 45.24	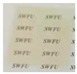	2.35 ± 0.15
ST5	2.09 ± 0.13	76.35 ± 12.74	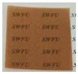	4.46 ± 0.02
ST10	2.20 ± 0.47	240.51 ± 71.88	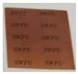	4.19 ± 0.05
ST15	2.01 ± 0.07	56.65 ± 7.65	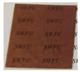	5.10 ± 0.10
ST20	2.04 ± 0.22	229.95 ± 41.52	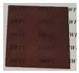	5.37 ± 0.14

Notes: mean values ± standard deviation.
